# A New Paradigm in Earth Environmental Monitoring with the CYGNSS Small Satellite Constellation

**DOI:** 10.1038/s41598-018-27127-4

**Published:** 2018-06-08

**Authors:** Christopher S. Ruf, Clara Chew, Timothy Lang, Mary G. Morris, Kyle Nave, Aaron Ridley, Rajeswari Balasubramaniam

**Affiliations:** 1 Climate and Space Dept., University of Michigan, Ann Arbor, MI USA; 2 University Corporation for Atmospheric Research, Boulder, CO USA; 3 NASA Marshall Space Flight Center, Huntsville, AL USA; 4 Jet Propulsion Laboratory, California Institute of Technology, Pasadena, CA USA; 5 Applied Defense Solutions, Columbia, MD USA

## Abstract

A constellation of small, low-cost satellites is able to make scientifically valuable measurements of the Earth which can be used for weather forecasting, disaster monitoring, and climate studies. Eight CYGNSS satellites were launched into low Earth orbit on December 15, 2016. Each satellite carries a science radar receiver which measures GPS signals reflected from the Earth surface. The signals contain information about the surface, including wind speed over ocean, and soil moisture and flooding over land. The satellites are distributed around their orbit plane so that measurements can be made more often to capture extreme weather events. Innovative engineering approaches are used to reduce per satellite cost, increase the number in the constellation, and improve temporal sampling. These include the use of differential drag rather than propulsion to adjust the spacing between satellites and the use of existing GPS signals as the science radars’ transmitter. Initial on-orbit results demonstrate the scientific utility of the CYGNSS observations, and suggest that a new paradigm in spaceborne Earth environmental monitoring is possible.

## Introduction

Traditional Earth orbiting satellites used for scientific studies and weather monitoring tend to be large and expensive, with a mass of hundreds to thousands of kg and a cost of hundreds of millions of USD. Recent examples include the NOAA Joint Polar Satellite System’s JPSS-1 (2200 kg and $655 million) launched in 2017^1^ and the NASA Global Precipitation Measurement core observatory (3850 kg and $933 million) launched in 2014^2^. These are highly capable platforms which carry multiple instruments and support a wide variety of science investigations. However, their large size and cost limit the number simultaneously flown to just one or a few. As a result, the time between measurements made at the same location can be several days or longer. This limits their ability to capture rapidly changing weather systems such as hurricanes, extreme rain events, and flooding on a regular basis. Temporal sampling can be significantly improved by flying a constellation of satellites that are well distributed, so that one of them will pass over every location more often. Doing so in a cost-constrained mission requires that the size, mass and complexity of each satellite be significantly reduced. A particular challenge is to make these reductions while retaining sufficient quality so the measurements made are still of scientific value.

The Cyclone Global Navigation Satellite System (CYGNSS) is the first NASA Earth science mission to use a constellation of small satellites. Its primary science objective is the measurement of wind speed in hurricanes and tropical cyclones with sufficient frequency to capture the rapid changes that occur when the storms intensify. The goal is a better understanding of the physical processes that cause hurricanes to form and develop, which can lead to improved forecasts of their location, strength and size^3^. Measurements are made of Global Positioning System (GPS) navigation signals reflected from the Earth surface. The signals are generated at an L-band frequency of 1.575 GHz in order to avoid attenuation and scattering by clouds or rain. In addition to the measurement of wind speed in hurricanes^4^, reflected GPS signals are also found to contain information about the moisture content of land surfaces^5^. Measurement of low moisture levels is of value for agricultural and meteorological applications, and the presence of high levels of moisture can be a good indicator of flood inundation after extreme rain events. Measurements are made using a new type of radar remote sensing which relies on the constellation of existing GPS satellites as the transmitter half of the radar^6^. CYGNSS provides only the receiver half of the radar on each of its satellites, which significantly reduces their complexity and cost^7^. In addition to carrying only a radar receiver, the satellite design itself is also simplified – most notably by using no active propulsion^8^. This presents significant challenges in orbital maintenance since the constellation needs to be well dispersed to provide the necessary temporal sampling. In comparison to the large, traditional Earth science satellites, the constellation of eight CYGNSS satellites has a combined mass of 198 kg and a total mission cost of $150 million.

## Results

### Differential Drag Orbit Configuration

All eight satellites in the CYGNSS constellation were launched on 15 December 2016 into a nearly circular 520 km altitude orbit on a single Pegasus XL rocket. The satellites were released individually by a deployment module attached to the third stage of the rocket. After deployment, the final velocity of each satellite is the vector sum of its deployment velocity and the orbital velocity of the rocket. The speed and direction of each deployment was adjusted so that the satellites after release had slightly different orbit speeds. Orbit speed determines orbit altitude, with faster satellites assuming a lower altitude. In this way, the separation between satellites will grow over time. In the early days after launch, vertical separations of several km were established due to their different orbit altitudes and horizontal separation between satellites continued to grow at rates of 30–300 km day^−1^ due to their relative speeds.

Spatial and temporal sampling properties of the science measurements made by the constellation are improved by spreading the satellites out around their orbit plane. This happens naturally due to their horizontal separation rates until the separation grows to one half the orbit circumference, after which the separation starts to decrease again as the faster moving satellite approaches and then “laps” the slower one. (There is minimal danger of collision because of their different average altitudes.) This cycle will repeat indefinitely if no adjustments are made to their relative speeds. Orbit adjustments to satellites are usually made via active propulsion systems they carry on board. In the case of the CYGNSS constellation, one primary design goal was to maximize the number of satellites in order to make measurements as frequently as possible. To that end, active propulsion was not included to reduce the per-satellite cost. However, attitude control (changes to the yaw, pitch and roll orientation of the satellite) was included in order to point the science antennas toward the Earth surface. Attitude control also allows for the technique of differential drag to be used to make adjustments to the satellites’ altitude and speed.

**Figure 1:**
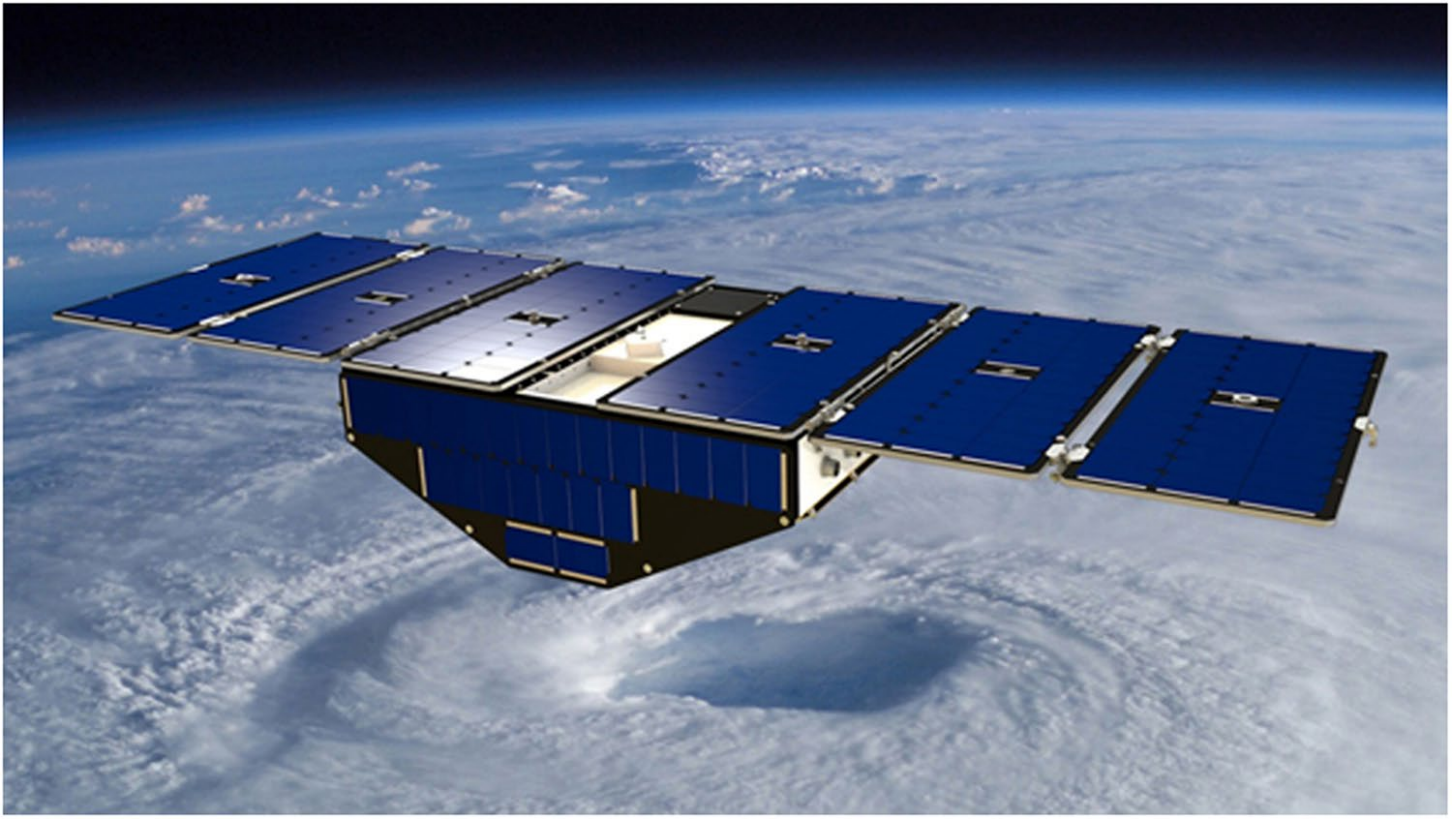
One of eight CYGNSS spacecraft in the constellation. Each spacecraft has a mass of 24.7 kg, requires 38 watts to operate in normal science data-taking mode, and has outer dimensions of 51 × 24 × 159 cm. The overall shape of the spacecraft is highly asymmetric, with long, wide, thin solar panels extending out to both sides of the central body. As a result, the atmospheric drag experienced by the spacecraft is highly dependent on its attitude.

A CYGNSS satellite is illustrated in Fig. 1. The spacecraft is shown in its nadir-pointed attitude, which it assumes when making science measurements. The orbital motion is from upper right to lower left in the figure, with the ram panel of the spacecraft’s central body facing forward and the large solar panels that extend to either side facing in the zenith direction. In this attitude, the solar panels present a minimum surface area in the direction of motion and atmospheric drag is minimized. When the spacecraft is pitched down by approximately 82°, the large solar panels will face in the direction of motion and atmospheric drag is maximized. The increase in drag causes a spacecraft’s altitude to decrease. When the pitch angle is returned to nadir pointing, the drag returns to its minimum state with the spacecraft now at a new (lower) orbit altitude and (higher) orbit speed. Such high drag maneuvers are performed periodically to adjust the relative velocity between spacecraft.

**Figure 2:**
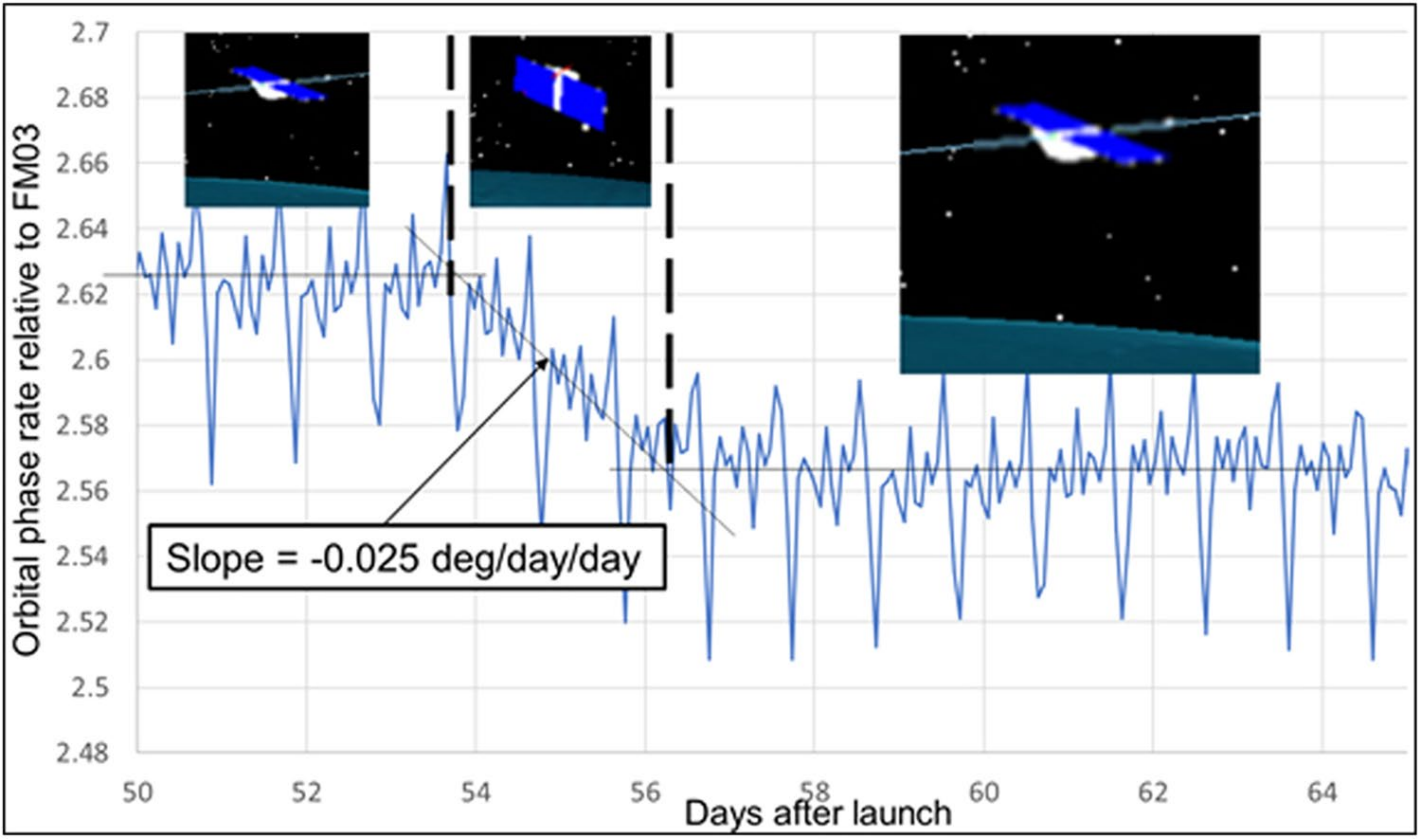
First “high drag” maneuver performed by one of the CYGNSS spacecraft. The attitude of the Flight Model 01 (FM01) spacecraft was pitched up to maximize atmospheric drag, lower its altitude, and raise its orbital velocity closer to that of another spacecraft (FM03) already at a lower altitude. This maneuver allows the rate-of-change of the spacing between spacecraft, or orbital phase rate, to be carefully controlled. The orbital phase rate is shown as a function of time (in units of days since launch) before, during and after the high drag maneuver.

An example of the impact a high-drag maneuver has on the rate at which the separation between two spacecraft changes is shown in Fig. 2. A time series plot of the orbital phase rate between two satellites is shown before, during and after a high drag maneuver lasting approximately two days. Orbital phase rate is the change in angular separation between the satellites around the orbit circumference, measured in degrees per day. In this case, the rate shown is between the highest and lowest altitude satellites in the constellation, Flight Model 01 (FM01) and FM03, respectively. Figure 2 shows short time scale variations in the phase rate due to non-uniformity in Earth’s gravity field, which affects orbital velocity, and also due to the eccentricity of the CYGNSS satellite orbits, which causes a slight increase in orbital velocity near perigee and decrease near apogee. Averaging across these variations, which occur on time scales of the 95 min orbit period, the longer term trend in phase rate is evident. Prior to the high drag maneuver, the mean orbital phase rate was ~2.62° day^−1^. In terms of distance, this corresponds to a change in separation of ~310 km day^−1^. A high-drag maneuver was initiated with FM01 on day 54 after launch. The increase in drag caused the satellite’s altitude to drop and its orbit velocity to increase toward that of FM03. After two days, FM01 was returned to its normal, nadir-pointed, attitude with a new phase rate of 2.57° day^−1^ and separation rate of ~305 km day^−1^ relative to FM03. This marked the first high drag maneuver performed during the CYGNSS mission. It demonstrated the feasibility of the method for constellation configuration maintenance and it showed the level of control that is possible for setting the phase rate and, ultimately, the spacing between satellites.

A detailed description of the differential drag maneuver and of plans for related mission operations are provided in Finley *et al*.^9^. Since the first attempt on 23 Feb 2017, numerous high-drag maneuvers have been performed, typically lasting between a few days and a couple of weeks each, to control the relative altitudes and velocities of the satellites. There have been several cases of conjunctions in angular separation by pairs of satellites, in which one passes over, or laps, the other. In those cases, the orbital phase rate will spread them apart again over time. The process is ongoing, with four of the eight satellites thusfar having been maneuvered into the same orbit altitude and period within their allotted spacing goals. A final constellation configuration is expected later in 2018 with the eight satellites dispersed approximately uniformly around the orbit circumference and all at the same altitude and with zero orbital phase rate. Regarding mission lifetime, the satellite in the lowest initial orbit altitude of 527 km has an estimated mission lifetime due to orbital decay of ~11 years. The other satellites, with initial orbit altitudes of 528–530 km, would have had somewhat longer lifetimes had they not been dragged down to a common altitude. Other factors, such as battery and solar panel degradation, are also expected to contribute to the ultimate mission life. It will likely be shorter than the lifetime of many larger satellites.

### Sampling Properties of the Full Constellation

Each CYGNSS satellite carries a 4-channel receiver that tracks and measures GNSS signals reflected by the Earth’s surface from at most four different GPS satellite transmitters. The full constellation of eight satellites thus can make up to 32 simultaneous measurements. In practice, there are usually more than 4 reflected GPS signals present within each receiver’s field of view, so 32 simultaneous measurements is the norm. These measurements are made nearly continuously, over both ocean and land, as the satellites orbit around the Earth. Measurements made over the ocean support science investigations related to surface wind and latent heat flux, and measurements over land support investigations related to soil moisture and flood inundation. The composite collection of measurements determines the constellation’s spatial coverage and sampling frequency, both of which are significantly enhanced by the large number of satellites.

Spatial coverage is determined by evaluating the fraction of the ocean surface between +/− 35° latitude sampled by CYGNSS within a specified time interval. NASA mission requirements call for this coverage to be above 70% within a 24-hour time period in order to adequately resolve the evolution of tropical cyclones throughout their life cycle. Two factors that drive the coverage statistic are the number of operational satellites and the spacing of those satellites along the orbital track.

**Figure 3:**
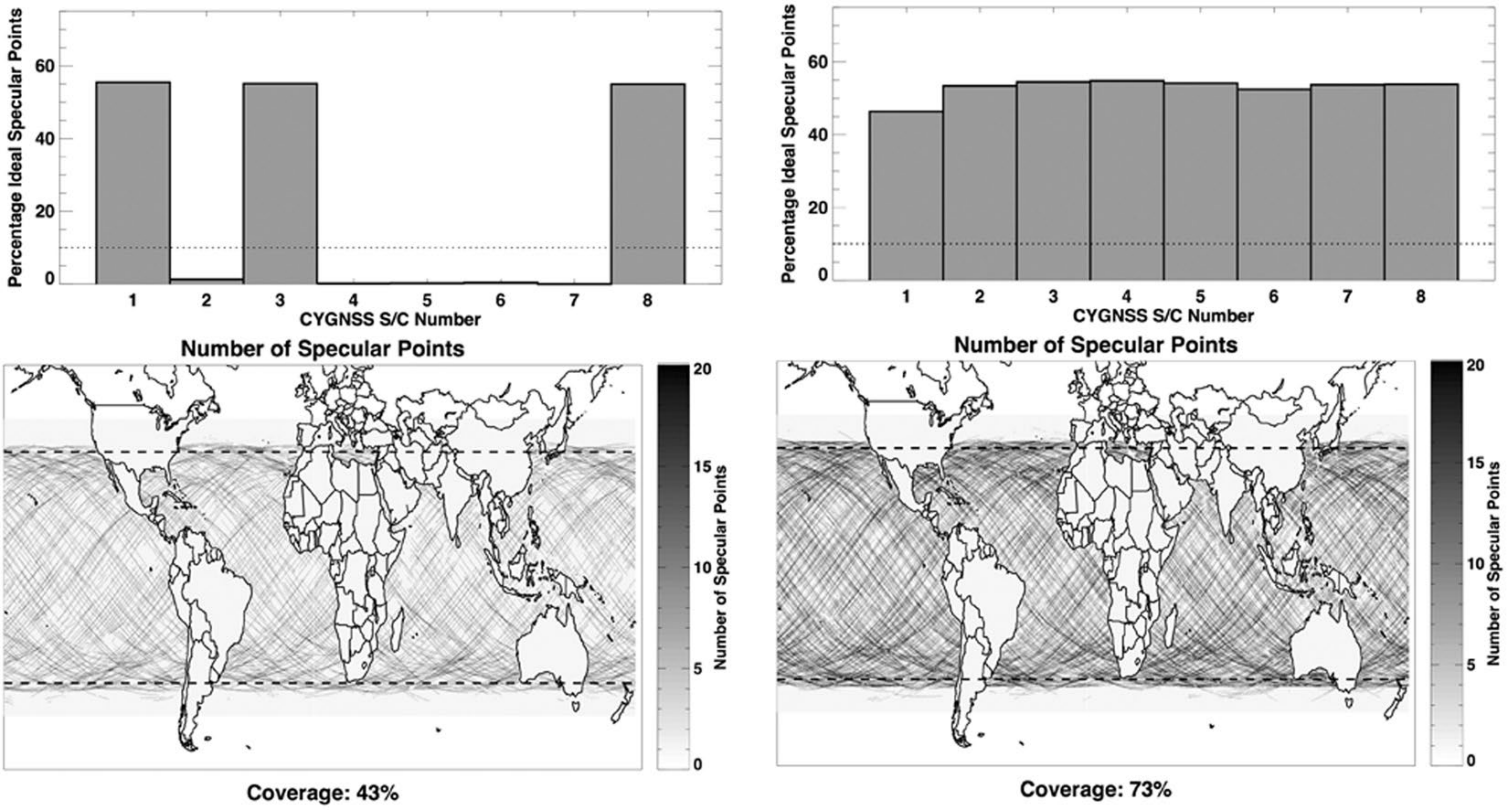
Science data coverage for April 13, 2017 (left) and September 10, 2017 (right). Top plots show the percentage of wind samples taken relative to the maximum possible number. Bottom plots show the distribution of samples across the Earth with a lat/lon resolution of 0.25 × 0.25°.

Figure 3 demonstrates how the number of satellites affects coverage. In the figure, the “Percentage Ideal Specular Points” represents the ratio between the actual number of science measurements and the maximum number possible if the satellite had been in science data-taking mode 100% of the time and every measurement taken had passed all science quality control tests. Early in the mission, the satellites were still being commissioned and most of them were not taking science data. For example, on 13 April 2017, only three of the eight CYGNSS satellites were in science mode. The left plots of Fig. 3 show that those three satellites produced 43% coverage. On 10 September 2017 (right plots of Fig. 3), all eight satellites were operating in science mode and 73% coverage was produced.

**Figure 4:**
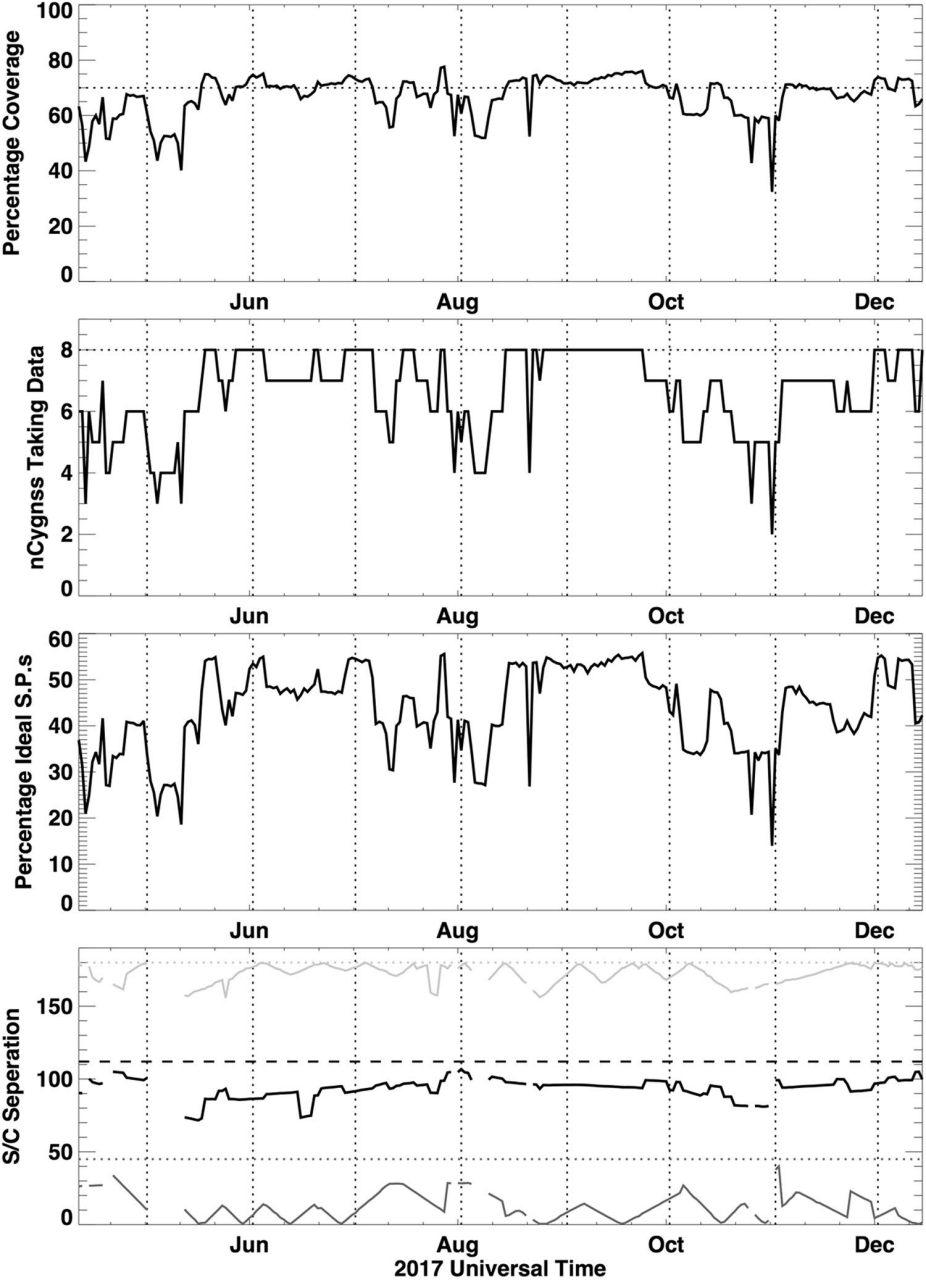
CYGNSS constellation coverage diagnostics during 2017. From top to bottom: the percentage coverage of the ocean between +/−35° latitude by the full CYGNSS constellation; the number of CYGNSS satellites producing at least 10% of the maximum number of measurements; the total percentage of the maximum number of measurements for the entire constellation; and the minimum (dark grey), mean (black), and maximum (light grey) satellite spacing of the satellites around the orbital plane in units of degrees (out of a full 360° orbit), with the expected values for a uniformly spaced constellation indicated by dashed and dotted lines.

While Fig. 3 shows the coverage for two very different days, Fig. 4 shows the coverage and related parameters as a function of time from April 2017 through the end of the year, with the beginning of each month marked by a vertical dashed line. The additional parameters are included to gain insight into what significantly affects the coverage. One such parameter is the number of satellites operating in science mode on any particular day. Beginning in May, engineering commissioning was completed and all eight CYGNSS satellites began operating in science mode most of the time. Subsequent interruptions in science mode resulted from a variety of reasons, including: (1) commanded high-drag maneuvers to properly space out the satellites; (2) commanded satellite attitude maneuvers to improve illumination of the solar panels when the angle between the orbital plane and the Earth-Sun vector was too high; and (3) occasionally, unexpected “safe mode” events when an anomaly is detected and non-essential systems (like science data taking) are automatically turned off until the mission operation center can evaluate the anomaly and command a return to science mode.

The vast majority of the variability in the coverage (top plot in Fig. 4) can be explained by the number of satellites in science mode (second plot) and the percentage of maximum possible measurements across the constellation (third plot). Indeed, the coverage and percentage of measurements have a correlation coefficient of 0.965. The inter-satellite spacing is not a significant determiner of coverage. In the bottom plot of Fig. 4, the three solid lines show the minimum, mean and maximum inter-satellite spacing between all eight satellites in the constellation. If any two adjacent satellites are close together, the minimum spacing is small. If two satellites are on diametrically opposite sides of the orbit, the maximum is 180°. For a uniformly distributed constellation, the spacing should be 45° between each adjacent pair of satellites. The dotted and dashed lines in the figure indicate the minimum (45°) and mean (112.5°) spacings for the uniform case. The maximum spacing would be 180°. The minimum and mean spacings are indicators of whether satellites are too close together to provide independent science measurements. For example, the minimum spacing is well above zero in early to mid September, meaning the constellation is well spaced out since no two satellites are close to one another. In late August, on the other hand, the minimum spacing is close to zero, indicating that at least two satellites are close together. However, the mean spacing generally remains between 70° and 100° throughout the year and the coverage is not significantly affected.

### Measurement of Ocean Surface Wind Speed

There is a long history of satellite measurements of ocean surface wind speed, beginning with early proof-of-concept missions in the 1970s and 80s^10,11^ and maturing into families of repeat missions used for extended climate studies and operational weather forecasting^12–14^. Current state-of-the-art measurement capabilities for the Special Sensor Microwave/Image (SSM/I) passive microwave sensors are 0.9 m s^−1^ uncertainty in wind speed with a spatial resolution of 25 km and revisit time of 2–3 days per satellite^15^. The capabilities of the QuikSCAT radar, which is no longer operational, were 0.9 m s^−1^ uncertainty in wind speed and 10° uncertainty in direction with a spatial resolution of 12.5 km and revisit time of 24 hr^16^. Notably, both of these instrument types operate at sufficiently high microwave frequencies that attenuation and scattering from rain can be significant and operation in extreme weather conditions such as mesoscale convective systems and tropical cyclones is severely limited^17,18^. Measurements of ocean surface wind speed at lower microwave frequencies with good penetration through heavy rain have been reported by the SMAP passive microwave sensor^19^. Its measurement uncertainty is 1.5 m s^−1^ with a spatial resolution of 40 km and revisit time of 3 days^20^. Notable in the case of SMAP is its coarser spatial resolution, which can limit its ability to resolve wind structure in the inner core of tropical cyclones.

Wind speed measurements are made by CYGNSS in a manner roughly analogous to that of previous spaceborne ocean wind sensing radars, by detecting changes in surface roughness caused by near surface wind stress^21^. Its wind speed measurement uncertainty is 1.4 m s^−1^ below 20 m s^−1^ and 17% above 20 m s^−1 ^^22^, with spatial resolution of 25 km^23^ and revisit time of 3 hr (median) and 7 hr (mean)^8^. The presence of 8 satellites in the CYGNSS constellation significantly reduces its revisit time relative to that of the individual satellites noted above. CYGNSS operates at a low microwave frequency of 1.575 GHz, close to that of SMAP, which enables it to penetrate through levels of precipitation up to and including that typically found in the inner core of tropical cyclones.

### Measurement of Ocean Surface Wind Speed in Hurricanes

**Figure 5:**
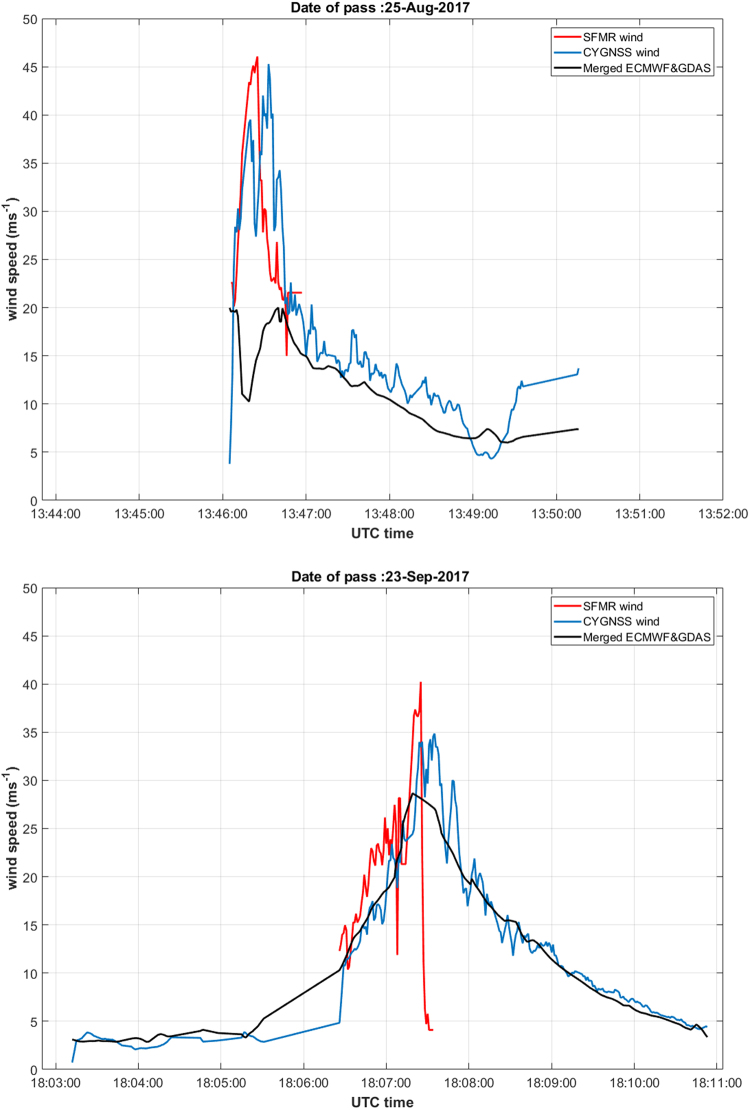
Coincident CYGNSS satellite overpasses and NOAA P-3 aircraft flights into hurricanes. CYGNSS measurements of surface wind speed are shown in blue. Coincident measurements by the SFMR instrument on the P-3 are shown in red for the overlapping portion of the airplane’s flight path. The black line shows the wind speed estimated by the ECMWF numerical weather prediction model. (top) Overpass of Hurricane Harvey on 25 August 2017 at 13:46–13:50 UTC. (bottom) Overpass of Hurricane Maria on 23 September 2017 at 18:03–18:10 UTC.

CYGNSS operated in science data-taking mode during almost all of the active 2017 Atlantic hurricane season and recorded many direct overpasses of its major storms. Some of the overpasses were coordinated with the NOAA Aircraft Operations Center which manages their fleet of hurricane hunter P-3 airplanes. The planes fly directly into hurricanes carrying specialized equipment designed to accurately measure wind speed at the ocean surface. To support the validation of CYGNSS measurements, some of the planes’ flights during 2017 were scheduled to coincide with the CYGNSS overpasses, resulting in near simultaneous measurements of surface wind speed in the inner core of the hurricanes. Two examples of this are shown in Fig. 5.

For the overpass of Hurricane Harvey on 25 Aug 2017 shown in Fig. 5a, the maximum winds measured by both CYGNSS and the Stepped Frequency Microwave Radiometer (SFMR) instrument on the P-3 can be seen to occur between 13:46:15–13:46:45 UTC^24^. The wind speeds are in general agreement over the time period of overlap. The coincident wind speed predicted by the numerical weather prediction (NWP) model of the European Centre for Medium-range Weather Forecasts (ECMWF), on the other hand, does not resolve the highest wind speeds near the storm center. This is typical of NWP models, which tend to be more accurate away from highly localized storm events. Figure 5b shows results for the CYGNSS overpass of Hurricane Maria on 23 Sep 2017. In this case, the NWP model does a better job of reproducing the storm center region, although the peak winds are still underestimated. The maximum winds measured by SFMR occur near 18:07:20 UTC. The abrupt drop in SFMR wind speed immediately after the maximum corresponds to penetration by the P-3 aircraft through the eyewall into the calm eye region. The CYGNSS measurement track did not fully enter the eye so its spatially averaged value for wind speed did not drop as low. Other cases of CYGNSS hurricane overpasses have demonstrated much larger decreases in wind speed in the eye region, but they were not accompanied by coincident P-3 underflights. CYGNSS coverage extends both before and after that of the P-3 to include a more complete sampling of the wind field on both sides of the storm center.

### Measurement of Ocean Surface Wind Speed under Heavy Precipitation

CYGNSS is able to measure ocean surface wind speed under heavy precipitation as a result of its low operating frequency, relative to other spaceborne wind sensors. It is also able to capture short-lived weather events such as convective storms due to the rapid sampling that results from use of a constellation of spacecraft. These two capabilities enable the study of wind patterns underlying tropical convective weather events.

By combining CYGNSS winds with Integrated Multi-satelliE Retrievals for Global Precipitation Measurement (IMERG) precipitation, we have found many examples of significant horizontal wind shear near convection. Hoover *et al*.^25^ performed a pre-launch study of simulated CYGNSS observations in the vicinity of tropical convection, which predicted that this horizontal shear would be associated with gust fronts driven by downdraft-induced outflows.

Hoover *et al*.^25^ found that these horizontal wind gradients near convection were easily observed when analyzing a contiguous track of specular points formed by surface reflections between a single CYGNSS observatory and a single GPS satellite. As a demonstration of this concept in real CYGNSS observations, we isolated and studied individual tracks that occurred during 26–30 August 2017. IMERG precipitation was linked to each CYGNSS specular point through a nearest-neighbor approach in space and by linear interpolation of precipitation in time. This created time series of precipitation along with matched wind speeds for each track.

**Figure 6:**
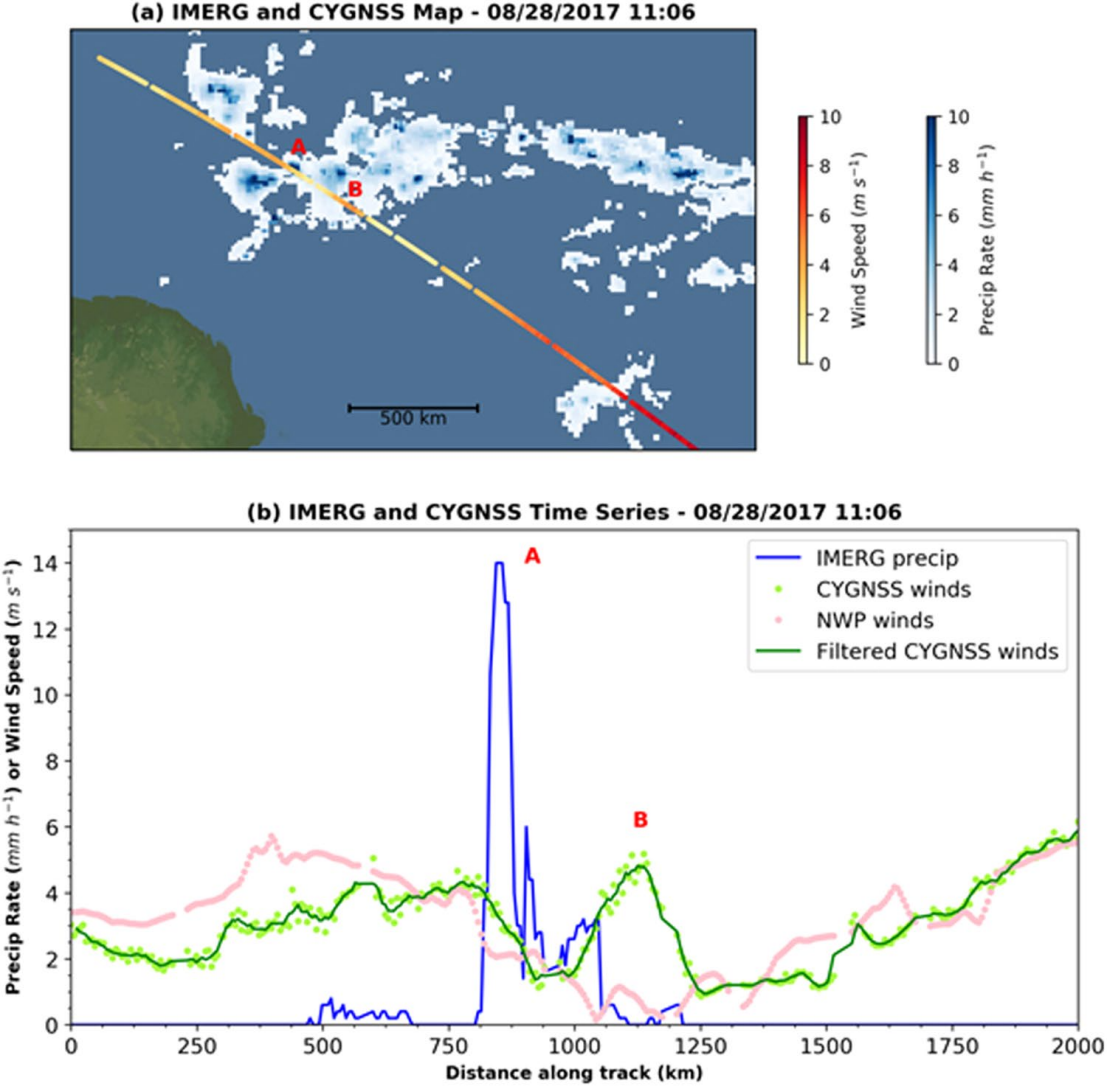
CYGNSS wind speed measurements across a strong convective storm. Start of track is 11:06 UTC on 28 August 2017, and direction of sampling is toward the southeast. Track is off northeast coast of Brazil. (top) Map view of CYGNSS with IMERG. A refers to cell with heavy precipitation. B refers to the apparent gust front. (bottom) Time series view. A and B labels are same as in (**a**). Filtered CYGNSS winds are created using a 5-point moving boxcar.

An example result is shown in Fig. 6. In this case a CYGNSS track passed directly through a cell (denoted by the A) embedded within mesoscale convection. Within the heaviest precipitation there is a decline in wind speed of ~2 m s^−1^. Then southeast of the main convective line (denoted by the B), wind speeds rapidly increase back to approximately 5 m s^−1^. This matches well the behavior predicted by Hoover *et al*.^25^ for CYGNSS observations of gust fronts near convection.

Interestingly, matched NWP model winds near this storm disagree significantly with CYGNSS winds within the apparent gust front. In order to study this more robustly, over the 5-day period, we performed a statistical analysis of matched CYGNSS and NWP winds both inside (0.2 M observations) and outside (2.6 M observations) IMERG-defined raining regions. Outside of rainfall, the root mean square error (RMSE) between CYGNSS and NWP was 2 m s^−1^ with zero bias, essentially matching CYGNSS pre-launch science requirements. However, where precipitation was above 0 mm h^−1^, the RMSE was instead 2.7 m s^−1^ with a CYGNSS bias of −0.1 m s^−1^.

Given that the change in bias is very small, thus ruling out significant attenuation (or amplification) of the reflected L-band GPS signal in precipitation, there are a couple potential explanations for this increased RMSE. It could occur if the NWP model was not placing convection of the right strength at the right location, relative to actual observations. Since CYGNSS provides a coupled measurement of wind speed and significant wave height, another possibility is that variability in wind-driven waves is increased near convection. Without correction for waves in the CYGNSS data, this could lead to increased differences between retrieved and model winds. There also could be a combination of the above effects. As the CYGNSS dataset grows in time, we will continue to study these convective wind observations, including analysis of time-varying winds in a specific location near convection, as well as analysis of 3D radar data (*e.g*., ground-based radar, GPM, etc.).

### Mapping of Inland Waterways

**Figure 7:**
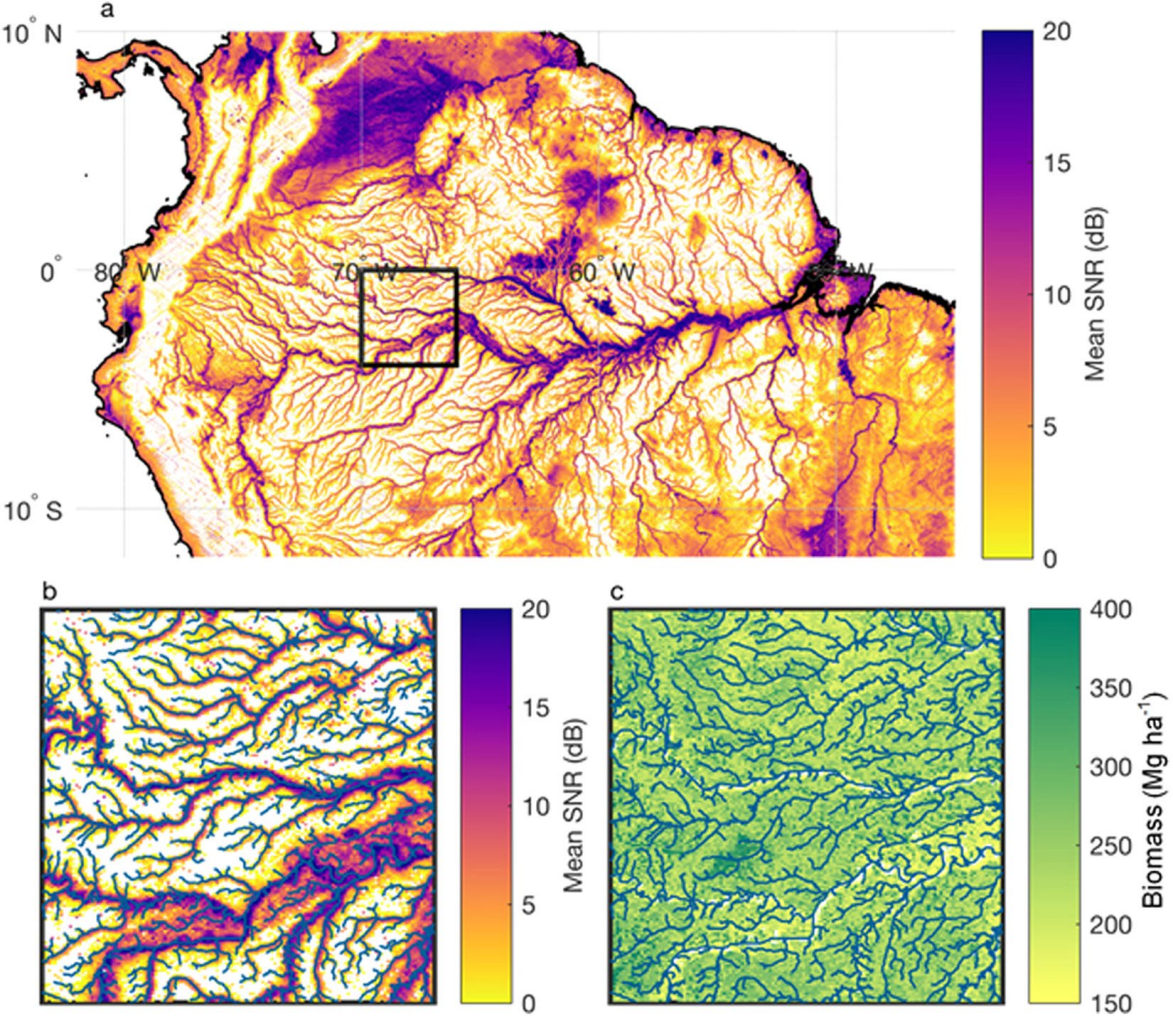
CYGNSS mapping of inland waterways in the Amazon. (**a**) Mean SNR, gridded to 3 km, over the Amazon for the time period 18 Mar–29 Dec 2017. Areas with surface elevation >600 m above sea level have been masked out due to a limitation in the CYGNSS on-board data compression algorithm. Corrective flight software was uplinked to the constellation in Dec 2017. (**b**) Inset of the black outlined box in (**a**), with river outlines from HydroSHEDS^38^ overlaid in green. (c) Biomass map^39^ for the black outlined box in (**a**), with river outlines from HydroSHEDS^21^ overlaid.

Observations of signal-to-noise ratio (SNR) from CYGNSS show the potential to map inland surface water. Figure 7 shows mean SNR over the Amazon and surrounding areas for the time period 18 Mar–29 Dec 2017. Higher SNR is observed over rivers and known wetland or seasonally inundated areas. River outlines produced by the Hydrological data and maps based on SHuttle Elevation Derivatives at multiple Scales (HydroSHEDS) are shown on top of the gridded SNR data in Fig. 7b. An increase of greater than 20 dB is seen over water, relative to surrounding areas.

This figure highlights two important aspects of the CYGNSS observations. First, SNR observations are sensitive to small (sub-kilometer) surface water features over land. Diffuse scattering of the GNSS signal from rough surface leads to a relatively large footprint on the surface (~15 × 15 km). Diffuse scattering often occurs over the ocean surface, and until recently it was thought that land surface reflections would be largely diffuse as well. However, flat surfaces such as water in small lakes or rivers produces specular scattering that is much stronger than the diffuse scattering from surrounding areas and so dominates the radar return, in which case the spatial footprint is approximately equal in size to the first Fresnel zone. For the CYGNSS measurement geometry, the first Fresnel zone is less than half a kilometer^26^, which explains why SNR observations are sensitive to the small Amazonian tributaries.

The second important aspect highlighted in Fig. 7 is that SNR observations are still sensitive to surface water even when the water is obscured by dense vegetation. Figure 7c shows a biomass map for part of the Amazon. In this region, biomass can be as high as 400 Mg ha^−1^. As long as there is surface water present, however, the observed SNR is still several dB greater than non-inundated areas.

### Imaging of Flooding Events

**Figure 8:**
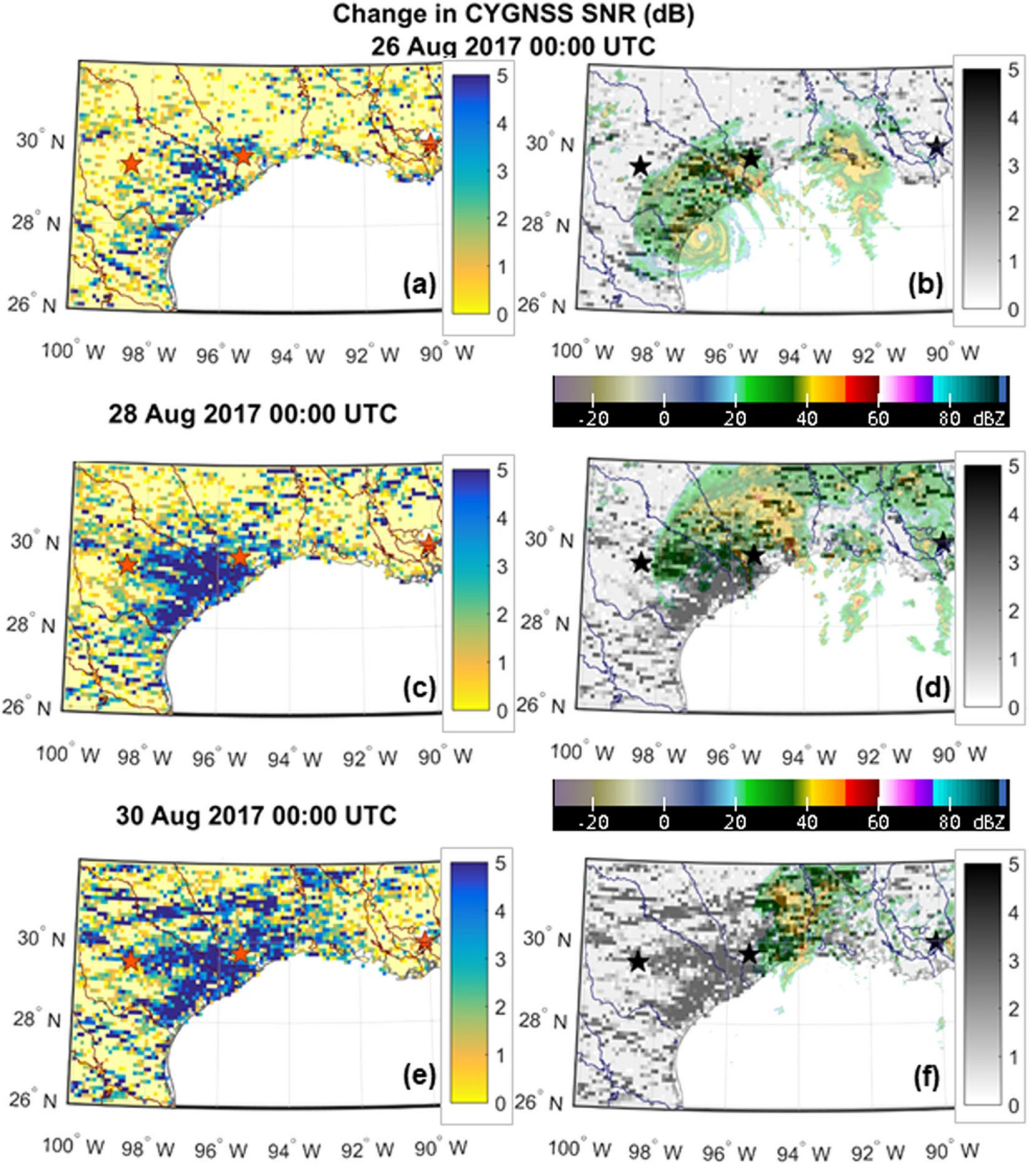
Changes in CYGNSS SNR observed during the Hurricane Harvey event. Data are visualized for (**a**,**b**) 26 Aug 2017, the day of landfall, (**c**,**d**) 28 Aug 2017, and (**e**,**f**) 30 Aug 2017. The left subplots (**a,c,e**) show CYGNSS data only. The right subplots (**b,d,f**) show the left subplots in gray scale, with a color overlay of composite NEXRAD radar imagery^40^ taken at the time stamps specified in the figure. Flood inundation is indicated by large increases in SNR, for example along the Gulf coast of Texas in the days after landfall.

The ability to detect inland waterways can be applied to rapidly changing circumstances during flooding events. This is illustrated in the series of images shown in Fig. 8 of CYGNSS SNR measurements over southeast Texas made shortly before and in the days after Hurricane Harvey made landfall on 26 Aug 2017. Harvey stalled as it made landfall, resulting in persistent and heavy rainfall across the region in the following days which led to major flooding. According to the Hurricane Harvey Tropical Cyclone Report produced by the National Hurricane Center^27^, “Harvey was the most significant tropical cyclone rainfall event in United States history, both in scope and peak rainfall amounts, since reliable rainfall records began around the 1880s.” In Fig. 8, flooding is indicated in the images by marked increases in the SNR from day to day. Note, in particular, the increase along the southeast Texas coast that spreads inland from 26 Aug to 28 Aug as the flood waters rise and spread. Radar imagery in the right-hand Fig. 8 subplots highlights both the path Harvey took as well as the areas where intense precipitation was prevalent. In Harvey’s wake, flooding persisted.

### Measurement of Near-Surface Soil Moisture

In addition to mapping of inland waterways and imaging of flooding, observations from CYGNSS also have the potential to monitor near-surface (0–5 cm) soil moisture. The L-band signals recorded by CYGNSS are similar to those utilized by satellites specifically designed for soil moisture remote sensing, such as European Space Agency Soil Moisture and Ocean Salinity (SMOS)^28^ and NASA Soil Moisture Active Passive (SMAP)^19^ satellites. L-band microwave signals are sensitive to changes in the dielectric properties of the soil, which primarily depend on its moisture content^29^. Soil with a higher moisture content will produce a stronger reflection than soil with a lower moisture content.

**Figure 9:**
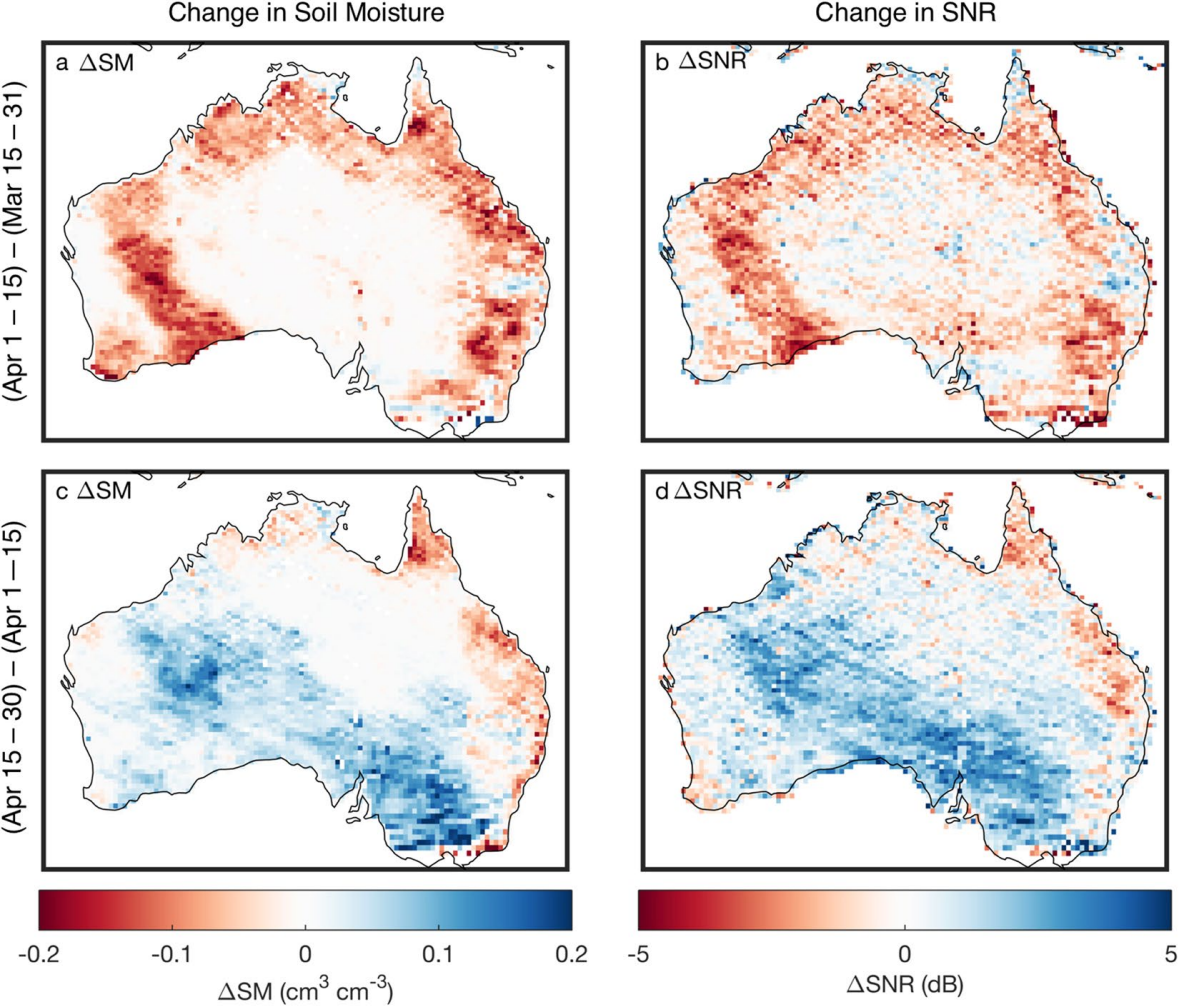
CYGNSS sensitivity to soil moisture. Change in mean SMAP soil moisture (**a**,**c**) from the last half of Mar to the first half of Apr 2017 (**a**) and the first half of Apr to the last half of Apr 2017 (**c**)^41^. (**b**,**d**) Same as (**a**,**c**) except for change in CYGNSS SNR.

Previous studies using a similar GNSS-R instrument onboard TechDemoSat-1 (TDS-1) showed sensitivity to soil moisture^30,31^, and observations by CYGNSS have similar sensitivities^32,33^. Figure 9 illustrates how temporal changes in soil moisture are accompanied by corresponding changes in SNR. Figure 9a,b show changes in soil moisture and SNR from the last two weeks of March to the first two weeks of April 2017 across Australia. Changes in each are considered in order to emphasize the underlying sensitivity of the measurement. A soil moisture retrieval algorithm would need to consider the value of SNR itself. Figure 9a indicates that the first half of April was drier than the last half of March for nearly all of Australia. Figure 9c,d show changes in soil moisture and SNR from the first two weeks of April to the last two weeks of April 2017. Figure 9c suggests that the mean soil moisture for the last two weeks of April was wetter than that in the first two weeks for the western and southern parts of Australia. In contrast, northeastern Australia continued to dry down. Changes in mean SNR for the same time periods (Fig. 9b,d) show similar spatial patterns of change as the soil moisture data, indicating that SNR observations are able to resolve the changes in soil moisture.

The data shown in Fig. 9 have been gridded to the 36-km native resolution of the SMAP soil moisture product to aid in a side-by-side comparison with CYGNSS. However, the native spatial resolution of the CYGNSS data over land is significantly better. CYGNSS and future GNSSS-R spaceborne observations thus have the potential to provide near-surface soil moisture information with better spatial resolution than the current state of the art.

## Discussion

The CYGNSS mission demonstrates that a constellation of small, low-cost satellites is able to make valuable contributions to Earth science and applications. The satellites use a simplified design that allows eight of them to be built and flown for significantly less than the typical cost of a single scientific satellite. One significant simplification is the lack of active propulsion. With no propulsion, the positioning and spacing between satellites is instead managed by adjusting spacecraft attitude, which changes the atmospheric drag and relative orbital velocity between satellites.

The use of a constellation of satellites allows for much more frequent measurements to be made than would be possible with a single satellite. This is especially useful when sampling infrequent or short-lived weather events such as storms and flooding. The frequency and distribution of samples is found to be fairly insensitive to the precise spacing between satellites, so long as they are not extremely close together. This relaxes the difficulty with maintenance of the constellation configuration and simplifies the complexity of mission operations.

The science payload on each CYGNSS satellite is a radar receiver which measures GPS navigation signals scattered by the Earth surface. The radar measures the strength of the scattered signal, which varies depending on the roughness and dielectric properties of the surface. Over the ocean, the sensitivity to roughness is used to estimate wind speed near the surface. Wind speed measurements are possible in the inner core of hurricanes and tropical cyclones, and under heavy precipitation. Over land, the sensitivity to dielectric properties makes possible the detection of inland waterways, flooding and near surface soil moisture. In each of these cases, previous satellites have exhibited similar measurement capabilities. However, the ability to make all of these measurements with a single instrument, the high density and frequency of samples made possible by a constellation of satellites, and the low overall cost of the mission make CYGNSS unique.

It should be possible to expand upon these capabilities in add-on or follow-on missions. An add-on mission might, for example, supplement the existing constellation with additional spacecraft to improve global coverage and revisit time. Use of a higher, or polar, inclination orbit would expand coverage to higher latitudes and could enable cryospheric science investigations. For example, measurements made in polar orbit by the TDS-1 technology demonstration satellite demonstrate the ability of the GNSS-R method to measure sea ice draft with high precision^34^. A follow-on mission might enhance the capabilities of the current CYGNSS receivers with technology advancements to measure other GNSS-R signals such as those transmitted by Galileo or other navigation satellites, as well as wider bandwidth signals transmitted by both GPS and Galileo. The use of wider bandwidth signals would improve the spatial resolution of ocean surface wind measurements and would also improve the ranging performance used for GNSS-R ocean altimetry^35^.

The scientific value of CYGNSS measurements, and possible applications in hurricane forecasting and storm surge and flood prediction, have not yet been fully realized. Many challenges still lie ahead for this new remote sensing method before it can be accepted as a reliable and well-understood measurement technique. Accurate calibration of the raw GPS measurements, proper interpretation of the calibrated measurements as indirect measurements of geophysical parameters, and integration of those measurements into meteorological and hydrological numerical prediction models are all ongoing tasks that will be refined and matured over time. In the future, it is hoped that small satellite constellations will become an essential, effective and efficient component of our spaceborne Earth monitoring capability.

## Methods

Measurements made by the CYGNSS satellites are converted from raw data to values of the scattering cross section of the Earth surface using a calibration methodology^7,36^. The scattering cross section values are converted to measurements of ocean surface wind speed using a retrieval algorithm described^7,23^. Data over land are processed slightly differently than over the ocean. Coherent reflections over the land surface are assumed. The peak of each delay-Doppler map (DDM) is normalized to the noise floor and then corrected for effects from antenna gain, range and transmit power, as described by

1 $$SNR=10\,\mathrm{log}({{\rm{\Gamma }}}_{rl})\propto 10\,\mathrm{log}(\frac{{P}_{rl}^{c}{({R}_{ts}+{R}_{sr})}^{2}}{N{G}^{r}{G}^{t}{P}_{r}^{t}})$$

where Γ_*rl*_ is the reflectivity of the surface, ${P}_{rl}^{c}$ is the coherently reflected power, *R*_*ts*_ is the distance between the transmitter and specular reflection point on the surface, and *R*_*sr*_ is the distance between the specular reflection point on the surface and the CYGNSS receiver, *N* is the noise floor of the DDM, *G*^*r*^ is the gain of the receiving antenna, *G*^*t*^ is the gain of the transmitting antenna, and ${P}_{r}^{t}$ is the transmitted power of the GNSS signal. A more complete discussion of these terms is contained in Chew *et al*.^37^.

### Data Availability

All raw data, calibrated scattering cross section values, and ocean surface wind speed measurements are available to the public at the NASA Physical Oceanography Distributed Active Archive Center, or PO.DAAC (https://podaac.jpl.nasa.gov/CYGNSS). In addition to the data, the PO.DAAC site also provides detailed documentation explaining the algorithms used to process the data, any caveats or corrections to the data quality or availability, and state-of-health reports about the satellites and the science instruments on them.

## References

[1] NOAA, Joint Polar Satellite System, http://www.jpss.noaa.gov/ (2018).

[2] Skofronick-Jackson G (2016). The Global Precipitation Measurement (GPM) Mission for Science and Society. Bull. Amer. Meteor. Soc..

[3] Ruf CS (2016). New Ocean Winds Satellite Mission to Probe Hurricanes and Tropical Convection. Bull. Amer. Meteor. Soc..

[4] Katzberg SJ, Walker RA, Roles JH, Lynch T, Black PG (2001). First GPS signals reflected from the interior of a tropical storm: Preliminary results from hurricane Michael. Geophys. Res. Lett..

[5] Rodriguez-Alvarez, N. *et al*. Soil Moisture Retrieval Using GNSS-R Techniques: Experimental Results Over a Bare Soil Field. *IEEE Trans Geosci*. *Remote Sens*. **47**(11), 10.1109/TGRS.2009.2030672 (2009).

[6] Martin-Neira, M. A Passive Reflectometry and Interferometry System (PARIS): Application to ocean altimetry. *ESA J*. **17** (1993).

[7] Ruf, C. *et al*. *CYGNSS Handbook*(ed. Ruf, C.)ISBN 978-1-60785-380-0 (Michigan Pub., 2016b).

[8] Ruf C (2013). CYGNSS: Enabling the Future of Hurricane Prediction. IEEE Geosci. Remote Sens. Mag..

[9] Finley, T. *et al*. Techniques for LEO Constellation Deployment and Phasing Utilizing Differential Aerodynamic Drag. *Proc. AAS/AIAA Astrodynamics Specialist Conf*., **150**, 1397–1411, ISBN: 978-087703605-0, Hilton Head, SC (2013).

[10] Jones W (1982). The SEASAT-A satellite scatterometer: The geophysical evaluation of remotely sensed wind vectors over the ocean. J. Geophys. Res..

[11] Cardone V, Chester T, Lipes R (1983). Evaluation of SEASAT SMMR Wind Speed Measurements. J. Geophys. Res..

[12] Risien CM, Chelton DB (2006). A satellite-derived climatology of global ocean winds. Remote Sens. Environ..

[13] Brennan M, Hennon C, Knabb R (2009). The operational use of QuikSCAT ocean surface vector winds at the National Hurricane Center. Wea. Forecasting.

[14] Atlas, R. *et al*. A Cross-calibrated, Multiplatform Ocean Surface Wind Velocity Product for Meteorological and Oceanographic Applications, BAMS, 157–174, 10.1175/2010BAMS2946.1 (2011).

[15] Wentz FJ (1997). A Well-calibrated Ocean Algorithm for SSM/I. J. Geophys. Res..

[16] Ricciardulli L, Wentz FJ (2015). A Scatterometer Geophysical Model Function for Climate-Quality Winds: QuikSCAT Ku-2011. J. Atmospheric Oceanic Tech..

[17] Hoffman RN, Leidner SM (2005). An Introduction to the Near-Real-Time QuikSCAT Data. Weather and Forecasting.

[18] Meissner, T., Ricciardulli, L. & Wentz, F. All-weather wind vector measurements from intercalibrated active and passive microwave satellite sensors. *Proc. 2011 IEEE Int. Geoscience and Remote Sensing Symp*., Vancouver, BC, CA, 10.1109/IGARSS.2011.6049354 (2011).

[19] Entekhabi D (2010). The Soil Moisture Active Passive (SMAP) mission. Proc. IEEE.

[20] Meissner, T., Ricciardulli, L. & Wentz, F. J. Capability of the SMAP Mission to Measure Ocean Surface Winds in Storms, *BAMS*, 10.1175/BAMS-D-16-0052.1 (2017).

[21] Zavorotny V, Voronovich A (2000). Scattering of GPS signals from the ocean with wind remote sensing applications. IEEE Trans. Geosci. Remote Sens..

[22] Ruf, C., Gleason, S. & McKague, D. S. Assessment of CYGNSS Wind Speed Retrieval Uncertainty. *IEEE J. Sel. Topics Appl. Earth Obs. Remote Sens*., 10.1109/JSTARS.2018.2825948 (2018).

[23] Clarizia, M. P. & Ruf, C. S. Wind Speed Retrieval Algorithm for the Cyclone Global Navigation Satellite System (CYGNSS) Mission. *IEEE Trans. Geosci. Remote Sens*. **54**(8), 10.1109/TGRS.2016.2541343 (2016).

[24] Uhlhorn EW (2007). Hurricane surface windmeasurements from an operational stepped frequency microwave radiometer. Mon. Wea. Rev..

[25] Hoover KE (2017). Use of an End-to-End-Simulator to analyze CYGNSS. J. Atmos. Oceanic. Technol..

[26] Katzberg, S. J. & Garrison, J. L. Utilizing GPS to Determine Ionospheric Delay over the Ocean. *NASA Technical Memorandum TM-475*0, 1–16. 10.1. 1.31.3748 (1996).

[27] Blake, E. S. & Zelinsky, D. A. Tropical cyclone report: Hurricane Harvey. *NOAA/NH*C, 76 pp. https://www.nhc.noaa.gov/data/tcr/AL092017_Harvey.pdf (2018).

[28] Kerr YH (2001). Soil moisture retrieval from space: The Soil Moisture and Ocean Salinity (SMOS) Mission. IEEE Trans. Geosci. Rem. Sens..

[29] Dobson MC, Ulaby FT, Hallikainen MT, El-Rayes MA (1985). Microwave Dielectric Behavior of Wet Soil-Part II: Dielectric Mixing Models. IEEE Trans. Geosci. Remote Sens..

[30] Camps A, Park H, Pablos M, Foti G, Gommenginger CP (2016). Sensitivity of GNSS-R Spaceborne Observations to Soil Moisture and Vegetation. IEEE Journal of Selected Topics in Applied Earth Observations and Remote Sensing..

[31] Chew C (2016). Demonstrating soil moisture remote sensing with observations from the UK TechDemoSat-1 satellite mission. Geophysical Research Letters..

[32] Nghiem SV (2017). Wetland Dynamics Monitoring with Global Navigation Satellite System Reflectometry. J. Earth and Space Science..

[33] Zuffada, C., Chew C. & Nghiem, S. V. GNSS-R algorithms for wetlands observations. *Proc. IEEE IGARSS, Fort Worth, TX*. 10.1109/IGARSS.2017.8127155 (2017).

[34] Li W (2017). First spaceborne phase altimetry over sea ice using TechDemoSat-1GNSS-R signals. Geophys. Res. Lett..

[35] Clarizia, M.P., Ruf, C., Cipollini, P. & Zuffada C. First Spaceborne Observation of Sea Surface Height Using GPS Reflectometry. *Geophys. Res. Let*., **43**, 10.1002/2015GL066624 (2016).

[36] Gleason S, Ruf C, Clarizia MP, O’Brien A (2016). Calibration and Unwrapping of the Normalized Scattering Cross Section for the Cyclone Global Navigation Satellite System (CYGNSS). IEEE Trans. Geosci. Remote Sens..

[37] Chew C (2017). SMAP radar receiver measures land surface freeze/thaw state through capture of forward-scattered L-band signals. Remote Sensing of Environment..

[38] Lehner B, Verdin K, Jarvis A (2008). New global hydrography derived from spaceborne elevation data. Trans. EOS..

[39] Avitabile V (2016). An integrated pan-tropical biomass map using multiple reference datasets. Glob. Chang. Biol..

[40] Iowa Environmental Mesonet. GIS NEXRAD Composites. Iowa State University. Subset used: August 2017–September 2017, accessed 13 February 2018, https://mesonet.agron.iastate.edu/docs/nexrad_composites/ (2018).

[41] O’Neill PE, Chan S, Njoku EG, Jackson T, Bindlish R (2016). SMAP Enhanced L3 Radiometer Global Daily 9 km EASE-Grid Soil Moisture, Version 1. NASA National Snow and Ice Data Center Distributed Active Archive Center, Boulder, Colorado USA.

